# Epinephrine auto injector administration by parents or patients for anaphylaxis during supervised oral food challenges and assessment of confidence

**DOI:** 10.1186/1710-1492-10-S2-A2

**Published:** 2014-12-18

**Authors:** Ingrid Baerg, Angela Alexander, Tiffany Wong, Timothy Teoh, Kyla Hildebrand, Sara Leo, Joanne Yeung, John Dean, Edmond Chan

**Affiliations:** 1 Division of Allergy and Immunology, Department of Pediatrics, BC Children’s Hospital, University of British Columbia, Vancouver, BC, V6H 3V4, Canada

## Background

Barriers to administering epinephrine auto injectors include failure to recognize signs and symptoms of anaphylaxis, administering oral antihistamines or asthma inhalers due to lack of education, failing to administer epinephrine correctly, fear of giving a needle, auto injector misplaced, and past experiences with spontaneous recovery. The aim of this study is to assess the impact of supervised auto injector administration by parents/patients on confidence, knowledge and skill for future treatment of severe allergic reactions.

## Methods

Patients with confirmed IgE-mediated food allergy at BC Children’s Hospital (2013-14), aged 2-17 years undergoing a physician supervised oral food challenge were approached and participation was voluntary. A pre- challenge questionnaire on patient and caregiver background information, confidence in recognizing a severe allergic reaction, and knowledge/skill in using an epinephrine auto-injector was completed by each participant. If an auto injector was deployed during the challenge, a post encounter questionnaire was administered to participants and practitioners collecting similar variables and qualitative data.

## Results

There have been 39 participants in this ongoing study. Mean age was 7.3 years (SD 4.3). 87% of parents were university educated, 26% health professionals, and 64% reported English as their first language. Parents ranked their child’s food allergy as severe (2.74/3.00, 95%CI, 2.39-3.10), but had only experienced using an auto injector 0.28 (95%CI, 0.10-0.46) times in the past. Confidence levels with auto injector use were 3.31/5.00 (95%CI,2.91-3.71) and skill levels 2.51/5.00 (95%CI,2.13-2.90). Parents who were health professionals had almost twice as much confidence and skill as non-health professionals (p<0.05). There was no statistically significant difference in confidence based on the number of times participants had experienced a severe reaction or used an auto injector in the community. Post challenge, 4 participants have required epinephrine, all administered by a parent.

## Conclusions

Our data suggest a moderate level of confidence but suboptimal skills in epinephrine auto injector use, influenced by whether parents are health professionals.

